# Effect of saffron supplementation on oxidative stress parameters: A systematic review and meta‐analysis of randomized placebo‐controlled trials

**DOI:** 10.1002/fsn3.2463

**Published:** 2021-08-05

**Authors:** Mojgan Morvaridzadeh, Shahram Agah, M. Dulce Estêvão, Ava Sadat Hosseini, Hafez Heydari, Omid Toupchian, Shima Abdollahi, Emma Persad, Ahmed Abu‐Zaid, Gholamreza Rezamand, Javad Heshmati

**Affiliations:** ^1^ Songhor Healthcare Center Kermanshah University of Medical Sciences Kermanshah Iran; ^2^ Colorectal Research Center Iran University of Medical Sciences Tehran Iran; ^3^ Universidade do Algarve Escola Superior de Saúde Faro Portugal; ^4^ Department of Education and Health Promotion School of Health Iran University of Medical Sciences Tehran Iran; ^5^ Cellular and Molecular Research Center Sabzevar University of Medical Sciences Sabzevar Iran; ^6^ Department of Nutrition and public Health School of Health North Khorasan University of Medical Sciences Bojnurd Iran; ^7^ Department for Evidence‐Based Medicine and Evaluation Danube University Krems Krems Austria; ^8^ Department of Pharmacology College of Graduate Health Sciences University of Tennessee Health Science Center Memphis TN USA

**Keywords:** malondialdehyde, oxidative stress, saffron, total antioxidant capacity

## Abstract

Oxidative stress (OS), the absence of equilibrium between prooxidants and antioxidants in the body, has been shown to play a pivotal role in the initiation and progression of many diseases. Saffron has been noted for its antioxidant capacity and can be used to improve OS parameters in unhealthy patients. Our aim was to evaluate the efficacy of saffron supplementation on OS parameters in unhealthy patients in randomized controlled trials (RCTs). We searched Medline, EMBASE, Cochrane CENTRAL, Scopus, and Web of Science without language restrictions for RCTs up until April 2021. Studies were included if they compared any form of saffron supplementation to placebo or no supplementation on OS parameters in unhealthy patients. Using a random‐effects model with calculated standardized mean difference (SMD) and 95% confidence intervals (CI), we quantitatively synthesized the data. Heterogeneity was assessed using Cochrane's *I* ^2^ values. Ten randomized controlled trials were eligible for this review. Seven were included in the meta‐analysis and indicated an association between saffron intake and a statistically significant decrease in malondialdehyde (MDA) levels (SMD: −0.40; 95% CI: −0.63, −0.17; *I* ^2^ = 32.6%) and a significant increase in total antioxidant capacity (TAC, SMD: 0.24; 95% CI: 0.05, 0.42; *I* ^2^ = 00.0%). Saffron intake was shown to significantly impact MDA and TAC, indicating its beneficial properties in improving OS in unhealthy patients. However, additional RCTs are required to evaluate the effect on other OS parameters.

## Graphical abstract

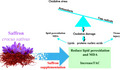

**Abbreviations**
4‐HNE: 4‐hydroxynonenal
AGE: advanced glycation end product
BMI: body mass index
CAT: catalase
CI: confidence interval
DPPH: diphenyl pycryl hydrazyl
FBG: fasting blood glucose
GPx: glutathione peroxidase
GR: glutathione reductase
GSH: glutathione
HbA1c: Hemoglobin A1C
IQR: interquartile range
MAPK: mitogen‐activated protein kinase
MDA: malondialdehyde
NADPH: nicotinamide adenine dinucleotide phosphate
NO: nitric oxide
Nrf2: the nuclear factor erythroid 2–related factor 2
OS: oxidative stress
ox‐LDL: oxidized *low‐density lipoprotein*
RCTs: randomized clinical trials
RNS: reactive nitrogen species
ROS: reactive oxygen species
SD: standard deviations
SE: standard errors
SMD: standard mean difference
SOD: superoxide dismutase
TAC: total antioxidant capacity
XO: xanthine oxidase

## 1 INTRODUCTION

Oxidative stress (OS) describes a microenvironment manifested by an absence of equilibrium between prooxidants and antioxidants (Liguori et al., 2018). The most important prooxidant enzymes comprise xanthine oxidase (XO), nicotinamide adenine dinucleotide phosphate (NADPH) oxidase, and nitric oxide synthase (NOS), whereas the most important antioxidant enzymes are glutathione reductase (GR), glutathione peroxidase (GPx), superoxide dismutase‐1 (SOD‐1), and catalase (CAT, Akbari et al., 2020; Maciejczyk et al., 2018). The repercussion of OS is the development of a biologically destructive microenvironment enriched with free radicals, namely reactive oxygen species (ROS) and reactive nitrogen species (RNS, Phaniendra et al., 2015). These free radicals can be generated in response to various endogenous sources, such as XO, NADPH, and NOS enzymes, and exogenous sources, such as pollution, medication, and radiation (Phaniendra et al., 2015). These free radicals play critical physiological roles in cell signaling, immune defense, and energy extraction, and their deleterious effects are adequately counterbalanced by anti‐oxidant machineries (Genestra, 2007). However, unneutralized excessive free radicals culminate in OS, and consequently bring about tissue injury and cell death (Cooke et al., 2003). Biochemically, the most important OS damage products comprise malondialdehyde (MDA), 4‐hydroxynonenal (4‐HNE), and advanced glycation end product (AGE, Maciejczyk et al., 2018). Total antioxidant capacity (TAC) and total oxidant status (TOS) are the two most frequent biochemical parameters employed to gauge the overall oxidant/anti‐oxidant profile (Maciejczyk et al., 2018).

Current research highlights the pivotal, pathophysiologic role of OS in the initiation and progression of a wide array of disorders, such as hypertension (Rodrigo et al., 2011), diabetes mellitus (Asmat et al., 2016), obesity (Manna & Jain, 2015), metabolic syndrome (Mahjoub & Masrour‐Roudsari, 2012), neurodegenerative disorders (Yaribeygi et al., 2018), cardiovascular disorders (Taleb et al., 2018), and malignancies (Hayes et al., 2020). Many factors, namely genetic, environmental, and dietary, can modulate the interplay between OS and the aforementioned disorders (Morvaridzadeh et al., 2020). Indeed, diet composition can considerably influence the OS microenvironment through the intrinsic prooxidant or antioxidant properties of such dietary elements (Namazi et al., 2018; Vetrani et al., 2013). Several dietary constituents have been greenlighted as naturally occurring antioxidants, such as cranberries (Blumberg et al., 2013) and a variety of spices, including garlic, ginger, and turmeric (Yashin et al., 2017).

Saffron, also known as *Crocus sativus*, is a common Mediterranean spice plant. It is composed of four key ingredients, namely crocin, crocetin, picrocrocin, and safranal (Roshanravan et al., 2020). Previous meta‐analyses have illustrated the beneficial effects of saffron on select blood pressure, glycemic, lipid, metabolic, and anthropometric indices (Asbaghi et al., 2019; Giannoulaki et al., 2020; Pourmasoumi et al., 2019; Rahmani et al., 2019; Roshanravan et al., 2020). A key property of saffron is its antioxidant capacity (Boskabady & Farkhondeh, 2016; Mashmoul et al., 2013). Several randomized controlled trials were carried out to examine the effect of saffron supplementation on mitigating OS (Ebrahimi et al., 2019; Hamidi et al., 2020; Karimi‐Nazari et al., 2019). However, the OS outcomes were inconsistent, as well as methodologically limited by small sample size, variable study duration, and heterogeneous underlying disorder. To the best of our knowledge, to date, no meta‐analysis has been performed to pool evidence from randomized controlled trials (RCTs) to inform concrete dietary recommendations concerning saffron supplementation and OS mitigation.

Thus, the goal of this research was to carry out a systematic review and meta‐analysis of all RCTs that evaluated the impact of saffron supplementation on OS indices in unhealthy individuals.

## 2 METHODS

### 2.1 Data sources and search strategy

This systematic review and meta‐analysis was performed according to The Preferred Reporting Items for Systematic Reviews and Meta‐Analyses (PRISMA) guidelines (Moher et al., 2009). Searches were run in Medline, EMBASE, Cochrane CENTRAL, Scopus, and Web of Science without language restrictions for RCTs up until April 2021. Crosschecking references of relevant reviews was also performed. The main terms searched in the databases included: “Saffron OR Crocus sativus OR Safranal OR Crocin OR Crocetin OR Picrocrocin AND Oxidative Stress OR Total Antioxidant Capacity OR antioxidant OR Oxidant OR reactive oxygen species OR Catalase OR Oxygen Radical Absorbance OR reactive nitrogen species OR lipid peroxide OR Malondialdehyde OR Nitric oxide”. Details on the search strategy and syntaxes for individual databases can be found in Appendix 1.

### 2.2 Selection criteria

We included randomized, placebo‐controlled or crossover design trials that compared any form of saffron supplementation versus no supplementation on OS parameters, namely TAC, MDA, GSH, GPx, SOD, NO, CAT, and isoprostanes, in unhealthy patients. Papers reporting sufficiently on outcomes comprised the difference in means with 95% confidence intervals (95% CI). Non‐randomized studies (cross‐sectional, case series, case studies, case–controls, and cohort studies) were excluded. We also excluded nonrandomized articles, abstracts, letters to the editor, in vitro and animal studies, and studies with less than one week of follow up after intervention. Table 1 indicates the Cochrane's evidence‐based PICO search criteria for this meta‐analysis.

**TABLE 1:** PICO inclusion criteria

Domain	Selection criteria
Participants	Unhealthy adults >18 years old with any disease
Interventions	Supplementation with any kind of saffron (Saffron OR Crocus sativus OR Safranal OR Crocin OR Crocetin OR Picrocrocin)
Comparators	Placebo No intervention
Outcomes	Oxidative stress parameters, including TAC, MDA, GSH, GPx, SOD, NO, CAT, and isoprostanes
Study design	Randomized controlled trials (including parallel or crossover studies)

Abbreviations: CAT, catalase activity; GPx, glutathione peroxidase activity; GSH, glutathione; MDA, malondialdehyde; NO, nitric oxide; SOD, superoxide dismutase; TAC, total antioxidant capacity.

### 2.3 Data extraction and statistical analysis

We extracted author details, general population characteristics, the dose of saffron, the kind of saffron supplement given, and main outcomes. Results were extracted in mean and standard deviation (*SD*). In order to calculate standard mean difference (SMD), results reported as standard error (SE), confidence interval, interquartile range (IQR), and the minimum and maximum value of each variable were converted to *SD*. We used STATA software version 11 (STATA Corp) and applied the random‐effects model. Statistical significance was defined at *p* < .05.

Heterogeneity was assessed using chi‐squared tests with significant heterogeneity considered at a *p*‐value <0.1 and *I* ^2^ statistic around 25% (*I* ^2^ = 25), 50% (I^2^ = 50), and 75% (*I* ^2^ = 75), which would mean low, medium, and high heterogeneity, respectively(Huedo‐Medina et al., 2006). Sensitivity analysis and funnel plots were used to evaluate the overall effect of each study. Funnel plots were used to visualize and evaluate publication error, and symmetry was examined using Egger's regression asymmetry test and Begg's rank correlation. Two researchers (JH and MM) independently performed risk of bias assessment using the Cochrane risk of bias tool (Higgins et al., 2011).

## 3 RESULTS

### 3.1 Search results

**FIGURE 1:**
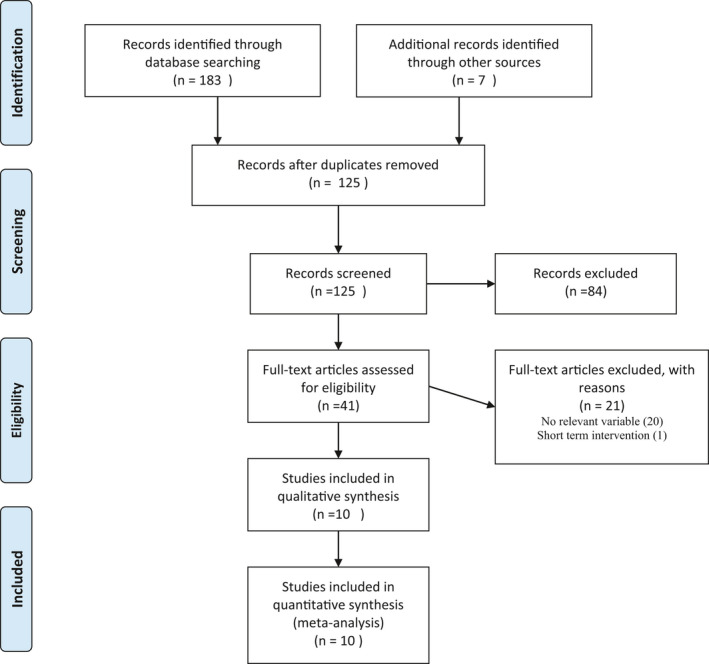
PRIMSA flow diagram

The initial search of electronic databases yielded a total of 183 studies. After removing duplicates, 124 unique records remained. After analyzing the titles and the abstracts, we concluded that 84 additional articles did not fulfil our inclusion criteria. After full text screening, we excluded further 20 studies as they failed to report on variables of interest. Thus, 10 trials met the established criteria to be included in this systematic review and meta‐analysis (Abedimanesh et al., 2020; Azimi et al., 2014; Ebrahimi et al., 2019; Ghaderi et al., 2019; Ghiasian et al., 2019; Hamidi et al., 2020; Karimi‐Nazari et al., 2019; Pour et al., 2020; Shahbazian et al., 2019; Tahvilian et al., 2020). The flow diagram of study selection is presented in Figure 1.

### 3.2 Characteristics of the included studies

Characteristics of the included trials are presented in Table 2. In total, 651 subjects were recruited in primary studies. Included articles were published between 2014 and 2020. The duration of supplementation with saffron ranged between four and twelve weeks. Saffron dosage ranged from 30 to 1,000 mg/day. The sample size of the included trials ranged from 40 to 80 subjects. All included trials were performed in Iran. The age of participants in included trials ranged from 31 to 57 years. The studies enrolled participants with type 2 diabetes (Azimi et al., 2014; Ebrahimi et al., 2019; Shahbazian et al., 2019), coronary artery disease (Abedimanesh et al., 2020), ulcerative sclerosis (Tahvilian et al., 2020), multiple sclerosis (Ghiasian et al., 2019), nonalcoholic fatty liver (Pour et al., 2020), rheumatoid arthritis (Hamidi et al., 2020), overweight/obese prediabetic patients (Karimi‐Nazari et al., 2019), and methadone maintenance treatment patients (Ghaderi et al., 2019). In addition, articles were performed on subjects with various baseline body mass index (BMI) values; nine studies (Abedimanesh et al., 2020; Azimi et al., 2014; Ebrahimi et al., 2019; Ghaderi et al., 2019; Giannoulaki et al., 2020; Hamidi et al., 2020; Karimi‐Nazari et al., 2019; Milne et al., 2015; Pour et al., 2020; Shahbazian et al., 2019; Tahvilian et al., 2020) were conducted on participants with a BMI over 25 kg/m^2^ up to 29.9 kg/m^2^, and one study did not report BMI (Ghiasian et al., 2019).

**TABLE 2:** Main characteristics of included studies

Study, (reference)	Country	Condition	Duration (weeks)	*n*	Group	Dose	Female gender, *n* (%)	Age, years, Mean ± *SD*	BMI, kg/m^2^, Mean ± *SD*	Main outcomes^a^
Abedimanesh et al. (2020)	Iran	Coronary artery disease	8	22 20	Saffron Placebo	30 mg/day 30 mg/day	NR NR	54.83 ± 5.99 56.00 ± 5.67	28.77 ± 3.75 27.91 ± 2.69	↓ Ox‐LDL
Azimi et al. (2014)	Iran	Type 2 diabetes mellitus	8	42 39	Saffron Placebo	1,000 mg/day	26(61.9) 24 (61.5)	57.02 ± 1.0 53.64 ± 1.3	28.86 ± 0.2 28.40 ± 0.2	↔ F2‐isoprostan
Ebrahimi et al. (2019)	Iran	Type 2 diabetes mellitus	12	40	Saffron	100 mg/day	20 (50)	55.2 ± 7.3	29.3 ± 4.9	↓MDA, ↔TAC
				40	Placebo	100 mg/day	24 (60)	53 ± 10.6	30.5 ± 4.7	
Karimi‐Nazari et al. (2019)	Iran	Overweight/obese prediabetic	8	36 39	Saffron Placebo	30 mg/day 30 mg/day	35 (64.9) 35 (64.4)	57.95 ± 8.12 57.9 ± 8.7	29.35 ± 1.50 28.78 ± 2.02	↑ DPPH
Tahvilian et al. (2020)	Iran	Ulcerative sclerosis	8	40	Saffron	100 mg/day	19 (47.5)	40.55 ± 12.71	26.95 ± 10.68	↔MDA, ↑TAC, ↑SOD, ↑GPX
				35	Placebo	100 mg/day	17 (48.6)	40.97 ± 11.34	24.80 ± 3.46	
Ghiasian et al. (2019)	Iran	Multiple sclerosis	4	20	Saffron	30 mg/day	17 (85)	29 ± 4.99	NR	↑TAC, ↓MDA, ↔TTG
				20	Placebo	30 mg/day	18 (90)	31.47 ± 5.31	NR	
Ghaderi et al. (2019)	Iran	Methadone maintenance treatment	8	26	Saffron	30 mg/day	NR	44.5 ± 9.4	24.5 ± 4.4	↑TAC, ↓MDA, ↔GSH
				27	Placebo	30 mg/day	NR	45.6 ± 9.9	25.2 ± 4.2	
Pour et al. (2020)	Iran	Nonalcoholic fatty liver disease	12	38	Saffron	100 mg/day	17 (44.7)	43.42 ± 10.62	28.85 (27.05, 32.68)^b^	↑TAC, ↓MDA
				38	Placebo	100 mg/day	16 (42.1)	42.05 ± 8.27	29.60 (27.99, 32.59)^b^	
Hamidi et al. (2020)	Iran	Rheumatoid arthritis	12	33	Saffron	100 mg/day	33 (100)	51.55 ± 8.26	28.17 ± 3.74	↔TAC, ↔MDA
				32	Placebo	100 mg/day	32 (100)	51.80 ± 9.62	28.39 ± 3.70	
Shahbazian et al. (2019)	Iran	Type 2 diabetes mellitus	12	32	Saffron	30 mg/day	24 (75)	53.5 ± 9.9	28.8 ± 4.0	↔TAC, ↔MDA
				32	Placebo	30 mg/day	21 (65.5)	52.4 ± 13	27.5 ± 4.2	

Abbreviations: GPX, glutathione peroxidase; GSH, total glutathione; MDA, malondialdehyde; *SD*, standard deviation; SOD, superoxide dismutase; T2D, type 2 diabetes mellitus; TAC, total antioxidant capacity; TTG, total thiol group; UC, ulcerative colitis.

^a^ Main outcomes were expressed in terms of statistical significance (p <.05) as either increased (↑), decreased (↓), or no difference (↔) between saffron versus placebo group.

^b^ Data were reported as median (interquartile range)

### 3.3 Qualitative results

Several OS‐related variables were assessed in the included trials, however, due to the small number of studies, they were not included in the meta‐analysis. The main results are summarized narratively. One study evaluated the effect of saffron intake on serum levels of oxidized low‐density cholesterol (ox‐LDL) as an OS indicator (Abedimanesh et al., 2020). Oxidized LDL is a marker of lipoprotein‐related OS and a risk factor for cardiovascular diseases (Holvoet et al., 2008). Abedimanesh et al. indicated that saffron intake significantly reduces ox‐LDL levels (Abedimanesh et al., 2020).

F2‐isoprostanes from arachidonic acid are the most primary substances of lipid peroxidation and are one of the most reliable variables for assessing OS according to recent investigations (Milne et al., 2015). One of the included studies investigated the effect of saffron intake on F2‐isoprostanes levels, and they reported that saffron intake had no significant effect on F2‐isoprostanes concentration (Azimi et al., 2014). Another finding of our primary studies was elevated levels of diphenyl pycryl hydrazyl (DPPH) radical scavenging activity after saffron intake, which can be explained by the fact that saffron can function as an antioxidant factor by donating a hydrogen atom to the DPPH radical anion (Karimi‐Nazari et al., 2019).

### 3.4 Meta‐analysis

**FIGURE 2:**
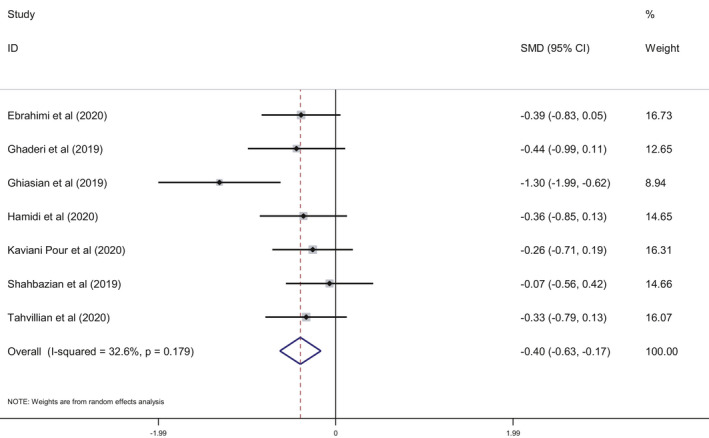
Forest plot showing the summary effect size for MDA levels between saffron and placebo groups

**FIGURE 3:**
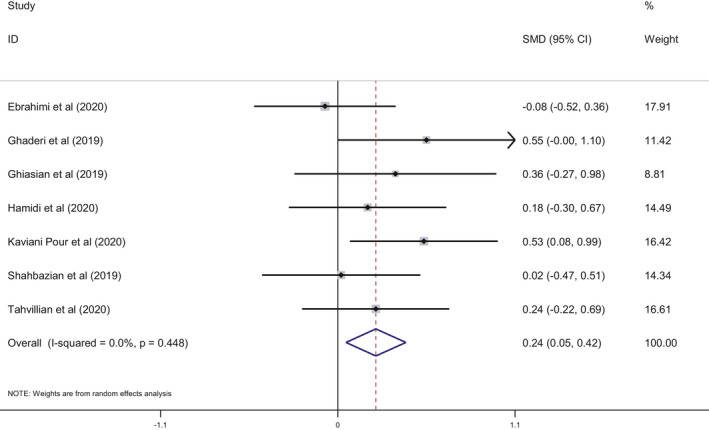
Forest plot showing the summary effect size for TAC levels between saffron and placebo groups

This meta‐analysis included seven studies and indicated that the saffron intake was associated with a statistically significant decrease in MDA levels (SMD: −0.40; 95% CI: −0.63, −0.17; *I* ^2^ = 32.6%, Figure 2) and a significant increase in TAC (SMD: 0.24; 95% CI: 0.05, 0.42; *I* ^2^ = 00.0%, Figure 3). We performed a subgroup analysis based on disease type (metabolic vs. nonmetabolic), duration and dose of saffron intake, and age of participants. For patients to be considered as having a metabolic disease, one of the indicators of metabolic syndrome needed to be met: hyperlipidemia, hyperglycemia, hypertension, and obesity. The results of the subgroup analysis showed that MDA decreased significantly in patients with nonmetabolic diseases and after a dose of over 50 mg/day compared to patients with metabolic diseases and doses of <50 mg/day. The subgroup analysis also revealed that saffron intake significantly increased TAC in nonmetabolic disease patients compared to metabolic patients. TAC was also significantly increased in patients in interventions of fewer than 10 weeks duration compared to longer interventions. Further, saffron intake also significantly increased TAC in middle‐aged adults compared to senior adults. Results of the subgroup analysis are presented in Table 3.

**TABLE 3:** Subgroup analysis assessing the effect of saffron intake on MDA and TAC

Variable	Sub‐grouped by		No. of arms	Effect size (SMD)	95% CI	*I* ^2^ (%)	*p* for heterogeneity
MDA	Disease type	Metabolic^b^	3	0.25	−0.52, 0.02	00.0	0.637
		Non‐Metabolic	4	**−0.55**	**−0.94, −0.17** ^a^	51.3	0.104
	Duration	≥10 weeks	4	**−0.28**	**−0.51, −0.04** ^a^	00.0	0.787
		<10 weeks	3	**0.64**	**−0.18, −0.10** ^a^	64.8	0.059
	Saffron dosage	≥50 mg/day	4	**−0.34**	**−0.56, −0.11** ^a^	00.0	0.980
		<50 mg/day	3	−0.57	−1.24, 0.10	75.8	**0.016**
	Age	Senior adults	3	**−0.28**	**−0.56, −0.01** ^a^	00.0	0.592
		Middle‐age adults	4	**−0.52**	**−0.92, −0.13** ^a^	56.5	0.075
TAC	Disease type	Metabolic^b^	3	0.16	−0.22, 0.54	50.1	0.135
		Non‐Metabolic	4	**0.31**	**0.05, 0.57** ^a^	00.0	0.776
	Duration	≥10 weeks	4	0.16	−0.11, 0.43	25.3	0.260
		<10 weeks	3	**0.36**	**0.06, 0.67** ^a^	00.0	0.690
	Saffron dosage	≥50 mg/day	4	0.21	−0.04, 0.47	16.9	0.307
		<50 mg/day	3	0.28	−0.04, 0.60	02.5	0.359
	Age	Senior adults	3	0.03	−0.24, 0.30	00.0	0.730
		Middle‐age adults	4	**0.41**	**0.16, 0.67** ^a^	00.0	0.774

Abbreviations: CI, confidence interval; MDA, malondialdehyde; SMD, standard mean difference; TAC, total antioxidant capacity.

^a^ Statistically significant.

^b^ According to metabolic syndrome components.

### 3.5 Quality appraisal

The results of the risk of bias assessment are presented in Appendix 2. Almost all included trials were rated at a low risk of bias for randomization, concealment, and blinding of participants and personnel. Further, five included studies were evaluated at an unclear risk for incomplete outcome data, and six of trials were rated at unclear risk for reporting bias. Three of included studies were rated unclear for other potential sources of biases.

## 4 DISCUSSION

To the best of my knowledge, this is the first comprehensive systematic review and meta‐analysis that investigated the impact of saffron supplementation on OS parameters. Saffron is widely used for many purposes, including medicinal applications, due to its complex composition of flavoring, aromatic, and colorful substances that are associated with health‐promoting benefits. Saffron compounds with higher biological activity include crocin, crocetin, picrocrocin, and safranal (Melnyk et al., 2010). In addition to the broad ancient uses of saffron for medicinal purposes, several studies have demonstrated that saffron has antioxidant, anti‐inflammatory, anticancer, antidepressant, and antiplatelet effects which suggest that saffron may be a potential therapeutic agent in many diseases, such as neurodegenerative diseases, diabetes mellitus, atherosclerosis, cancer, and sexual dysfunction, among others (Christodoulou et al., 2015; Leone et al., 2018; Razavi & Hosseinzadeh, 2017; Schmidt et al., 2007; Shakeri et al., 2020).

The major components of saffron have been studied in animal and cellular models and have been shown to have positive effects on the level and activity of enzymes involved in cellular antioxidant activity, such as CAT and SOD. Meanwhile, the levels of recognized markers of OS, such as MDA (an indicator of the peroxidation of lipids), were shown to be decreased after treatment with saffron constituents (reviewed in Lopresti & Drummond, 2014; Yousefi et al., 2020).

The total antioxidant status of an individual may also be evaluated by measuring the TAC, as initially proposed by Miller et al.,^1993^. This parameter is easily determined and can be applied in many clinical contexts to evaluate the TAC of foods, as many nutrients have been recognized by their role as antioxidants (reviewed in Kaliora & Dedoussis, 2007; Kusano & Ferrari, 2008). Our study evaluated the clinical context of the effect of saffron consumption on OS markers, and mainly focused on the results obtained for MDA and TAC levels. The seven trials included in the meta‐analysis indicated that the intake of saffron significantly decreased serum MDA and increased TAC (Figures 2 and 3), which highlights the positive effect that saffron appears to exhibit on OS.

Oxidative stress is associated with a wide range of diseases, such as diabetes mellitus and metabolic syndrome (including hyperlipidemia, hyperglycemia, hypertension, and obesity), which all of which are risk factors for cardiovascular diseases (Denisenko et al., 2020; Gil‐del Valle et al., 2005). To date, several studies have been carried out to evaluate the effect of saffron on different OS markers in these aforementioned diseases and others (Razavi & Hosseinzadeh, 2017; Pour et al., 2020). For example, in patients with nonalcoholic fatty liver disease, the ingestion of 100 mg saffron per day for 12 weeks induced a significant increase in TAC and decrease in MDA levels (Pour et al., 2020) while the intake of 15 mg of crocin twice a day for 8 weeks induced a significant reduction of the pro‐oxidant‐anti‐oxidant balance, in patients with metabolic syndrome (Nikbakht‐Jam et al., 2016).

Recent systematic reviews and meta‐analyses have highlighted a possible positive effect of saffron against cardiovascular diseases and as a modulator of serum lipid profile, showing that saffron can lead to decreases in serum total cholesterol and triglyceride concentrations, as well as an increase in high‐density lipoprotein levels. However, these results were based on a limited number of trials and should be interpreted with caution (Asbaghi et al., 2019; Pourmasoumi et al., 2019; Rahmani et al., 2019; Roshanravan et al., 2020).

Several other studies have shown that saffron or its biologically active components may have a positive impact on several parameters related to glucose metabolism, in patients with different non‐communicable diseases, and in patients undergoing hypoglycemic treatment, despite some discrepancies in the obtained conclusions. For example, saffron intake was shown to decrease fasting blood glucose (FBG), fasting insulin, and, for longer interventions, decrease HbA1c in one study (Sohaei et al., 2019). However, in another analysis, the effect on FBG was similar but more significant for interventions carried out for periods of 12 weeks or more, while no significant effect was observed in HbA1c (Rahmani et al., 2020). The intake of crocin was also evaluated and may also reduce FBG (Naserizadeh et al., 2020). In addition, another recent meta‐analysis focusing solely on patients with diabetes mellitus or metabolic syndrome assessed the effects of either saffron or its component, crocin, on several outcomes related to the metabolic profile of the patients. The authors concluded that although saffron appears to be an important regulator of FBG, however, most trials carried out thus far are not robust enough to clearly state the impact of saffron on these patients (Giannoulaki et al., 2020). These contradictory results may be due to the fact that saffron components are difficult to absorb by the human body and act only in the gastrointestinal tract (Naserizadeh et al., 2020). It is clear in all reported results that all the findings need to be carefully interpreted as the different trials included present high levels of heterogeneity at several levels.

The results obtained after the subgroup analysis depicted statistically significant positive effects of saffron for both MDA and TAC levels in patients with non‐metabolic diseases. Additionally, when elevated doses of saffron were used (50 mg/day or more) or in shorter interventions (fewer than 10 weeks), similar results were observed for MDA and TAC levels, respectively (Table 3). In this analysis, the age of the participants was determined to be another important factor to consider when assessing the health benefits of saffron, as TAC levels increased for younger patients (middle age vs. senior adults, Table 3). Several possible mechanisms could be responsible for the beneficial effect of saffron on improving OS parameters. It has recently been shown that saffron may affect cardiometabolic outcomes through its effect on non‐coding RNAs, such as microRNAs (miRNAs, Ahmadi Khatir et al., 2018). MiRNAs are endogenous ∼23‐nucleotide RNAs that can bind to different locations on mRNAs of protein‐coding genes to modulate expression of these genes (Emami, Akbari, et al., 2019, Emami, Nekouian, et al., 2019; Talebi et al., 2020). MicroRNAs are also known to impact the stability and evolution of several mRNAs (Akbari et al., 2016; Zamani et al., 2019). It has also been demonstrated that saffron may affect up‐stream genes related to OS, such as Nrf2 and MAPK, through changes in miRNAs (Ashrafizadeh et al., 2020; Hassani et al., 2017).

Although the results on the effects of saffron against OS are promising, several factors should also be considered when interpreting the available data. Plants’ composition and quality vary with the methods used for cultivation, production, and extraction, affecting the possible outcomes and their reproducibility (Moratalla‐Lopez et al., 2021; Schmidt et al., 2007). Additionally, the means of saffron or its components consumption may also affect bioavailability, which may also affect the health benefits being evaluated. More studies are needed to fully understand how saffron and its constituents are absorbed and reach the cells to exert their effect (Giannoulaki et al., 2020).

Additional parameters that need to be taken in consideration include the amount of saffron ingested, the duration of the intervention, and the number of patients enrolled in each trial. The studies available and included in this work were all carried out with small groups of patients, weakening the statistical value of the results. As discussed by Kaliora and Dedoussis (Kaliora & Dedoussis, 2007), the characteristics of the studied patients (e.g., age, sex, ethnicity, body mass index, initial total antioxidant status, and diet consumed) should also be considered carefully. For example, this meta‐analysis only includes studies that were carried out in Iran, which limits the possible extrapolation of the results for other populations. Another limitation of this review is that we didn't register the protocol in advance. Further, when studying unhealthy subjects, different trials may be needed for different diseases. The stage of the disease, the medication administered, and additional factors that may contribute to improving the outcomes evaluated need to be identified so they do not interfere with the final results. More studies should be carried out addressing these questions to increase the current knowledge base about the health benefits of saffron on different illnesses.

## 5 COMPETING INTERESTS

The authors have no conflict of interest to declare.

## 6 ETHICS APPROVAL AND CONSENT TO PARTICIPATE

Not applicable.

## 7 CONSENT FOR PUBLICATION

All authors consent for publication of article in this journal.

## AUTHOR'S CONTRIBUTION

**Javad Heshmati**: Conceptualization, methodology, manuscript preparation **Mojgan Morvaridzadeh and Gholamreza Rezamand**: Data extraction, writing, original draft preparation. **Shahram Agah and Shima abdollahi**: Data analysis, investigation. **M. Dulce Estêvão:** Writing, original draft preparation, reviewing, and editing. **Emma Persad**: Writing, reviewing, and editing, final draft validation. **Ahmed Abu‐Zaid and Omid Toupchian**: Writing original draft. **Ava sadat Hosseini and Hafez Heydari:** Editing and revising the manuscript.

## Data Availability

Not applicable.
